# Immunogenicity, Effectiveness, and Safety of Inactivated Virus (CoronaVac) Vaccine in a Two-Dose Primary Protocol and BNT162b2 Heterologous Booster in Brazil (Immunita-001): A One Year Period Follow Up Phase 4 Study

**DOI:** 10.3389/fimmu.2022.918896

**Published:** 2022-06-09

**Authors:** Rafaella F. Q. Grenfell, Nathalie B. F. Almeida, Priscilla S. Filgueiras, Camila A. Corsini, Sarah V. C. Gomes, Daniel A. P. de Miranda, Adelina J. Lourenço, Olindo A. Martins-Filho, Jaquelline G. de Oliveira, Andrea Teixeira-Carvalho, Guilherme R. F. Campos, Mauricio L. Nogueira, Pedro Augusto Alves, Gabriel R. Fernandes, Leda R. Castilho, Tulio M. Lima, Daniel P. B. de Abreu, Renata G. F. Alvim, Thaís Bárbara de S. Silva, Wander de J. Jeremias, Dayane A. Otta, Ana Carolina Campi-Azevedo, Immunita-001 Team, Priscila Fernanda S Martins

**Affiliations:** ^1^ Diagnosis and Therapy of Infectious Diseases and Cancer, Oswaldo Cruz Foundation (Fiocruz), Belo Horizonte, Brazil; ^2^ Department of Infectious Diseases, College of Veterinary Medicine, University of Georgia, Athens, GA, United States; ^3^ Hospital da Baleia, Benjamin Guimarães Foundation, Belo Horizonte, Brazil; ^4^ Grupo Integrado de Pesquisa em Biomarcadores, Oswaldo Cruz Foundation (Fiocruz), Belo Horizonte, Brazil; ^5^ Laboratório de Imunologia Celular e Molecular, Oswaldo Cruz Foundation (Fiocruz), Belo Horizonte, Brazil; ^6^ Laboratório de Pesquisas em Virologia (LPV), Faculdade de Medicina de São José do Rio Preto (FAMERP)São José do Rio Preto, São José do Rio Preto, Brazil; ^7^ Hospital de Base, São José do Rio Preto, Brazil; ^8^ Department of Pathology, University of Texas Medical Branch, Galveston, TX, United States; ^9^ Imunologia de Doenças Virais, Oswaldo Cruz Foundation (Fiocruz), Belo Horizonte, Brazil; ^10^ Biosystems Informatics, Oswaldo Cruz Foundation (Fiocruz), Belo Horizonte, Brazil; ^11^ Cell Culture Engineering Laboratory (COPPE), Universidade Federal do Rio de Janeiro, Rio de Janeiro, Brazil; ^12^ Laboratório de farmacologia experimental, College of Pharmacy, Universidade Federal de Ouro Preto, Ouro Preto, Brazil

**Keywords:** SARS-CoV-2, COVID-19, vaccine, immune response, coronavac, BNT162b2, heterologous booster

## Abstract

**Background:**

Effective and safe vaccines against severe acute respiratory syndrome coronavirus 2 (SARS-CoV-2) are critical to controlling the COVID-19 pandemic and will remain the most important tool in limiting the spread of the virus long after the pandemic is over.

**Methods:**

We bring pioneering contributions on the maintenance of the immune response over a year on a real-life basis study in 1,587 individuals (18-90 yrs, median 39 yrs; 1,208 female/379 male) who underwent vaccination with two doses of CoronaVac and BNT162b2 booster after 6-months of primary protocol.

**Findings:**

Elevated levels of anti-spike IgG antibodies were detected after CoronaVac vaccination, which significantly decreased after 80 days and remained stable until the introduction of the booster dose. Heterologous booster restored antibody titers up to-1·7-fold, changing overall seropositivity to 96%. Titers of neutralising antibodies to the Omicron variant were lower in all timepoints than those against Delta variant. Individuals presenting neutralising antibodies against Omicron also presented the highest titers against Delta and anti-Spike IgG. Cellular immune response measurement pointed out a mixed immune profile with a robust release of chemokines, cytokines, and growth factors on the first month after CoronaVac vaccination followed by a gradual reduction over time and no increase after the booster dose. A stronger interaction between those mediators was noted over time. Prior exposure to the virus leaded to a more robust cellular immune response and a rise in antibody levels 60 days post CoronaVac than in individuals with no previous COVID-19. Both vaccines were safe and well tolerated among individuals.

**Interpretation:**

Our data approach the effectiveness of CoronaVac association with BNT162b2 from the clinical and biological perspectives, aspects that have important implications for informing decisions about vaccine boosters.

**Funding:**

Fiocruz, Brazil.

## Introduction

Group-level immunity acquired through vaccination is imperative to mitigate the effects of the COVID-19 pandemic (1). Among COVID-19 vaccines, the vaccine containing inactivated virus (CoronaVac) produced by Sinovac Biotech and Instituto Butantan is the most common delivered worldwide. The two-dose regimen of CoronaVac has been deployed globally in 39 countries (2). In phase 3 trials, the two-dose regimen of CoronaVac showed 50·7%, 65·9% and 83·5% vaccine effectiveness against symptomatic COVID-19 disease in Brazil (3), Chile (4) and Turkey (5).

The emergence of SARS-CoV-2 variants with increased infectivity and transmissibility worldwide and the waning of humoral responses, especially of neutralising antibodies, might lead to lower vaccine protection (6). A test-negative real life case control study in Brazil showed 55·0% and 34·7% effectiveness against symptomatic infection, and 82·1% and 72·5% against severe outcomes, respectively at 30- and 180-days post CoronaVac. Similar study revealed that a BNT162b2 booster improved protection in 92·7% against infection (7).

Hence, in this cross-sectional study, SARS-CoV-2-specific humoral and cellular response were evaluated together with effectiveness over a year in a group of healthcare workers with or without previous COVID-19 infection, who underwent vaccination with two doses of CoronaVac and BNT162b2 booster in Brazil. Complete evaluation of the immune response over time on real life basis is unknown and have important implications for informing decisions about vaccine boosters.

## Materials and Methods

### Clinical Recruitment and Sample Collection

We conducted a cross-sectional immunogenicity and safety study (Immunita-001) of two-dose regimen of CoronaVac (Sinovac/Butantan), followed by a heterologous booster dose of mRNA vaccine (BNT162b2, Pfizer/BioNTech). Workers from two hospitals in Belo Horizonte, Brazil (Hospital da Baleia; Hospital Metropolitano Dr Célio de Castro) who had received primary protocol in the first two months of 2021 were enrolled after had given informed consent. Booster dose was received 6 months later. Blood samples for immunogenicity were taken at months 1, 2, 3, 6, 9 and 12 post-second dose vaccination. Briefly, venous blood was drawn by venipuncture and centrifuged at 2000 g for 5 min. Participants were monitored during the study for suspected symptoms manifestation such as: fever, cough, shortness of breath, fatigue, myalgia, headache, loss of taste or smell, sore throat, congestion or runny nose, nausea, vomiting or diarrhea. COVID-19 suspected participants were tested by reverse transcription quantitative polymerase chain reaction (RT-qPCR) with nasopharyngeal swabs collected from 3 to 7 days of onset symptoms, followed by sequencing when positive on the molecular test. Adverse events on the first 15 days post vaccination were also reported.

### Outcomes

The primary outcomes were anti-spike IgG antibodies, serum virus neutralisation titers 50% (VNT50) against Delta and Omicron variants, and cytokines, chemokines, and growth factors quantification. Secondary outcomes included local and systemic reactogenicity profiles, and SARS-CoV-2 infection and hospitalization.

### Anti-SARS-CoV-2 Spike IgG Antibody Assay

Serum samples were tested for SARS-CoV-2 spike protein specific antibody of the whole study population. The protein of SARS-COV-2 was produced in stable recombinant HEK293 cells and antibody detection was measured by enzyme-linked immunosorbent assay (ELISA) (8), with some modifications. Briefly, ninety-six-well plates (Corning) coated with 50 µl/well of 200 ng of purified Spike protein in phosphate buffered saline (PBS), pH 7·4, were washed and blocked with 2% nonfat dry milk in PBS for 2 h. Diluted sera in blocking buffer (1:40) was added and plates were incubated for 2 h, followed by an incubation of 90 min with a 1∶60,000 dilution of the secondary antibody HRP-conjugated goat anti-human IgG (Sigma Aldrich). Reaction was initiated by the addition of 3,3′,5,5′-tetramethylbenzidine (Sigma Aldrich) for 15 min and stopped with 1N HCl. The optical density (OD) was read at 450 nm using the Multiskan FC plate reader (Thermo Fisher).

### Viral Microneutralisation Assay with Variants of Concern

A viral microneutralisation assay (VNT) for SARS-CoV-2 was performed for Delta (B.1.617) and Omicron (B.1.1.529) variants following an adaptation of previous protocols (9, 10). For this approach, serum samples from 150 vaccinated participants with no history of COVID-19 were randomly selected and were comparable to the study population regarding gender and age distributions. Thirty samples were distributed at each time point after CoronaVac administration (30, 60, 90, 180 and 270 days after the second dose, where the last time point also corresponded to 30 days after the booster dose of BNT162b2). Part of the data generated from this analysis related to 30-, 60- and 270-days post vaccination against Omicron variant was previously published (11). Correlations, however, are presented here for the first time. Samples were inactivated at 56°C for 30 min and submitted to a two-fold serial dilution (from 1:20 to 1:2560). For each sample, eight serum concentrations were incubated for 1h at 37°C with fresh media containing 50 TCID50/ml of live SARS-CoV-2 Delta and Omicron. After incubation, 100 µl of these solutions were transferred to 96 well plates seeded in the day before with 10^4^ Vero CCL-81 cells, and incubated for 72h, at 37°C and 5% CO2. Cells were fixed with 10% formaldehyde and stained with crystal violet. The neutralisation capacity was determined by the presence of cytopathic effect across the dilutions, and for each sample, it was obtained the reciprocal dilution value where 50% of viral cytopathic effect was avoided (VNT50). Each sample was tested in triplicate and VNT50 was calculated using the Spearman-Karber algorithm (12).

### Immune Soluble Mediators’ Quantification

A high-throughput 27-plex Luminex assay (Bio-Rad) was used for quantification of a range of immune soluble mediators in sera of 200 participants, including: chemokines (CXCL8; CCL11; CCL3; CCL4; CCL2; CCL5; CXCL10), pro-inflammatory cytokines (IL-1β; IL-6; TNF-α; IL-12; IFN-γ; IL-15; IL-17), regulatory cytokines (IL-1Ra; IL-4; IL-5; IL-9; IL-10; IL-13) and growth factors (FGF-basic; PDGF; VEGF; G-CSF; GM-CSF; IL-7; IL-2). Measurements were performed on a Bio-Plex 200 instrument (Bio-Rad).

### Identification of COVID-19 Positive Cases by RT-qPCR and Sequencing by Sanger and NGS

In case of suspected COVID-19, purified RNA was directed to RT-qPCR using the Charité/Berlin protocol (13), that detect the amplification of the SARS-CoV-2 E gene and the Center for Disease Control and Prevention (CDC-EUA) protocol that detect the amplification of two regions of SARS-CoV-2 N gene (N1 and N2). The RT-qPCR reaction was performed with the GoTaq^®^ Probe 1-Step RT-qPCR System kit (Promega). For Charité/Berlin protocol, was used 10 μL GoTaq Probe qPCR Master Mix with dUTP 2X (Promega), 0·4 μL GoScript RT Mix 1-step RT-qPCR (Promega), 2 μL 2019-nCoV gene E [Integrated DNA Technologies (IDT)], 0·4 μL of a 10 μM RNAse P forward and reverse primers (IDT), 0·3 μL of a 10 μM RNAse P probe (IDT) and 1·5 μL nuclease free water. For CDC protocol, was used 10 μL GoTaq Probe qPCR Master Mix with dUTP 2X (Promega), 0·4 μL GoScript RT Mix 1-step RT-qPCR (Promega), 1·5 μL primer/probe mix for N1, N2 or RP (2019-nCoV RUO Kit – IDT), 3·1 μL nuclease free water. Thermal cycling was performed at 45°C for 15 min, followed by 95°C for 2 min and 40 cycles of 95°C for 15 s and 60°C for 1 min. QuantStudio 12K Flex Real Time PCR system or ViiATM 7 Real Time PCR system was used and the results were analyzed using the Thermo Cloud platform. Individuals who became positive for COVID-19 were excluded from that point on from the population analysis to generate immunogenicity data. Positive samples with cycle threshold (Ct) <25 were sequenced by Sanger sequencing and positive samples with Ct<34 were sequenced by Next-generation sequencing (NGS). For Sanger data, the contigs were assembled using the sangeranalyzeR package in R with the parameter to trim at a Phred Score of 20. The sequencing libraries were prepared using the Illumina COVIDSeq Test Kit (Illumin) (14), including the addition of a set of custom primers (**Supplementary Table 1**). The libraries were sequenced in the Illumina MiSeq platform producing paired read of 150bp. The variant identification was performed by the ViralFlow pipeline (15). The genomic sequences were deposited in GISAID database with the names also described in **Supplementary Table 1**.

### Statistical Methods

Data analysis was performed using GraphPad Prism^®^ software for comparative analysis between two groups, carried out by Student *t*-test or Mann-Whitney test, according to the data distribution. Multiple comparisons were performed by ANOVA followed by Tukey’s test or Kruskal-Wallis test followed by Dunn’s post-test. The correlation between antibodies concentration was determined using Spearman (p<0·05). Signatures of soluble mediators were designed by first converting the serum levels, originally expressed as continuous variables (pg/mL) into categorical data (percentual proportion) using the global median values as the cut-off to identify the proportion of subjects with high levels of mediators. The proportion of subjects with increased levels (above 50th percentile, gray zone) was underscored. Comprehensive heatmap analysis was performed using the heatmap.2 function of the R software version 3.0.1 and gplots package. The heatmap was assembled based on the levels of mediators observed at each range. The constructs were assembled using the customized functions available from Bioconductor packages scaled from the 10th to the 90th percentile. Pearson and Spearman correlation analysis was used to assemble integrative mediator networks. The significant correlations (p<0·05) were select and employed to build networks using the open-source Cytoscape software platform. Mediators were clustered into 4 major groups, referring to chemokine (circle), pro-inflammatory cytokines (squares), regulatory cytokines (diamond) and growth factors (hexagons). The correlations “r” scores were employed to identify the connectivity involving strong (thick connecting edges) and moderate (thin connecting edges) between mediators. Positive and negative correlations were identified by continuous or dashed lines, respectively. The total connectivity between attributes, the number of attributes involved in the strong correlation neighborhood (counts) and the balance between pro-inflammatory/regulatory mediators were calculated for each time-point.

## Results

A total of 1,587 participants were consented and enrolled in the study and samples were collected from March to December 2021. During this period, Gamma variant was dominant from February/2021 to July/2021 while Delta variant was dominant from the end of July/2021 to January/2022 and Omicron from February/2022. Participants ranged in age from 18 to 90 years old (median 39 yrs). A total of 1,208 (76·1%) of the 1,587 participants were female and 457/1,587 (28·8%) reported pre-existing comorbidity (**Table 1**). The most common pre-existing comorbidities were hypertension, obesity, asthma, and diabetes, respectively present in 174 (11·0%), 95 (6·0%), 61 (3·8%) and 42/1587 (2·7%) of the participants.

**Table 1:** Demographic data of participants in the study.

Age, Years		
	n	%
18-40	998	62.9
41-60	560	35.3
61-90	29	1.8
**Gender**		
	n	%
Male	379	23.9
Female	1,208	76.1
**COVID-19 prior to vaccination**		
	n	%
Yes	273	17.2
No	1314	82.8
**Comorbidities**		
	n	%
Systemic arterial hypertension	191	12.0
Obesity	95	6.0
Asthma	63	4.0
Hypothyroidism	50	3.2
Diabetes	42	2.7
Chronic renal disease	6	0.4
Rheumatoid arthritis	6	0.4
Hyperthyroidism	4	0.3

Age, gender, COVID-19 prior to vaccination and comorbidities are presented in absolute number and percentage.

### Immunogenicity

**Figure 1:**
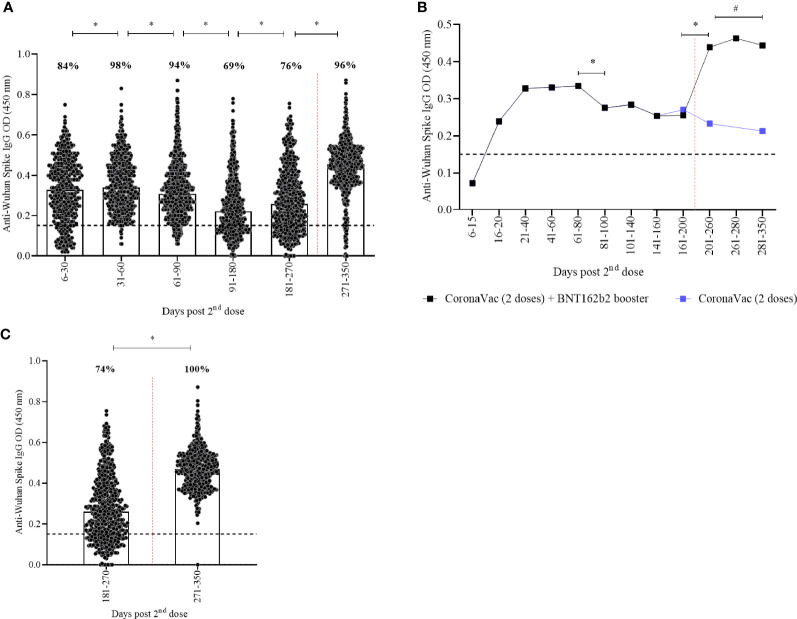
Kinetics of anti-spike IgG levels over a one-year period of CoronaVac in a two-dose primary protocol and BNT162b2 heterologous booster. Median OD of anti-spike IgG, measured by ELISA, is represented by bars, percentage values indicate seropositivity rate. The limit of detection of 0.1508 is represented by the black lines. Red dotted lines represent booster dose timepoint. In **(A, C)** individual points present each vaccinated participant. In **(B)** median of anti-spike IgG are shown. Mann-Whitney and ANOVA statistical differences are presented by letters for comparisons over timepoints (*) and comparison between recipients with or without the booster dose (#).

Significant differences in anti-spike IgG titers were seen in all timepoints (**Figure 1A**, p<0.05; **Supplementary Table 2**). A decline on IgG titers was seen 80 days post the second dose of CoronaVac and restored after the BNT162b2 booster dose (**Figures 1A, B**) which induced a 1·7x increase in comparison to the prior timepoint. Recipients who did not receive a booster dose remained 74% seropositive over a one-year period, although IgG titers remained lower in comparison to those who receive the booster dose (**Figure 1B**, p<0·001; **Supplementary Table 1**). Seropositivity rate was higher (98%) after 31-60 days and lower (69%) after 91-180 days after CoronaVac (**Figures 1A, C**). BNT162b2 booster dose increased seropositivity to 96%. When considering booster recipients with at least 15 days of vaccination, a seropositivity rate of 100% was observed (**Figure 1C**, p<0·001).

**Figure 2:**
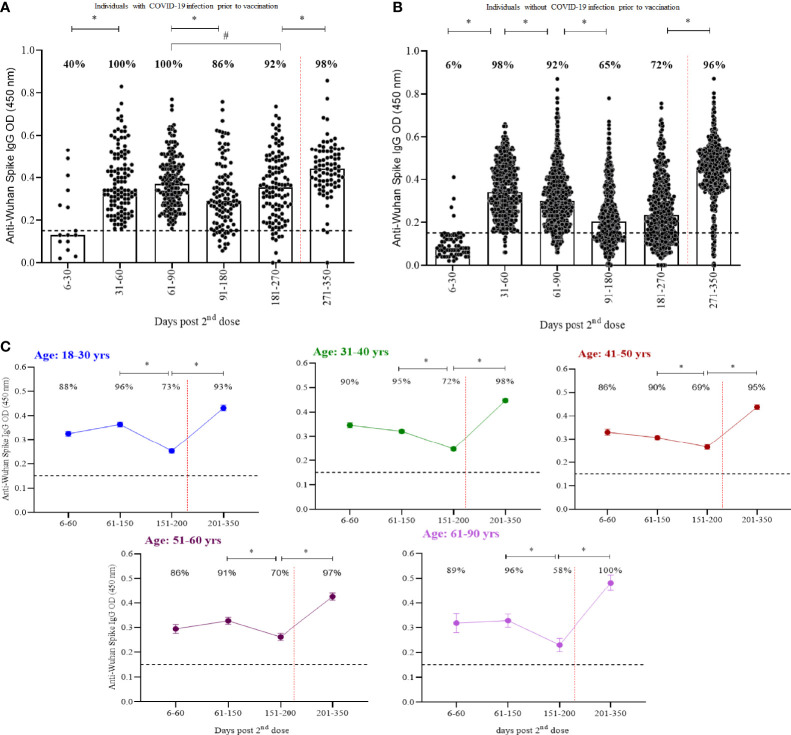
Kinetics of anti-spike IgG over a one-year period separated by **(A)** participants with COVID-19 infection prior to vaccination, **(B)** participants without COVID-19 and **(C)** age groups. Median OD of anti-spike IgG, measured by ELISA, is represented by bars, percentage values indicate seropositivity rate. Limit of detection of 0.1508 is represented by the black lines. Dotted red lines represent booster dose timepoint. Mann- Whitney and ANOVA statistical differences are presented by letters for comparisons over time points within the same groups (*) and between individuals with and without prior COVID-19 in the same time period (p < 0.0001) (#).

Two hundred seventy-three participants had RT-qPCR confirmed COVID-19 prior to vaccination and presented an increased seropositivity rate in comparison to those without previous infection (**Figures 2A, B**). Prior infection induced a rise in anti-spike IgG levels from 60 to 270 days post CoronaVac that were superior to those induced in previously uninfected individuals (p<0·0001). No significant differences were seen after booster. Similar responses were seen with anti-Spike IgG by age groups (**Figure 2C**; **Supplementary Table 2**). Seropositivity in older adults (61-90 years) was 15% lower than in younger adults (18-30 years) at days 150-200 post CoronaVac and restored after booster.

**Figure 3:**
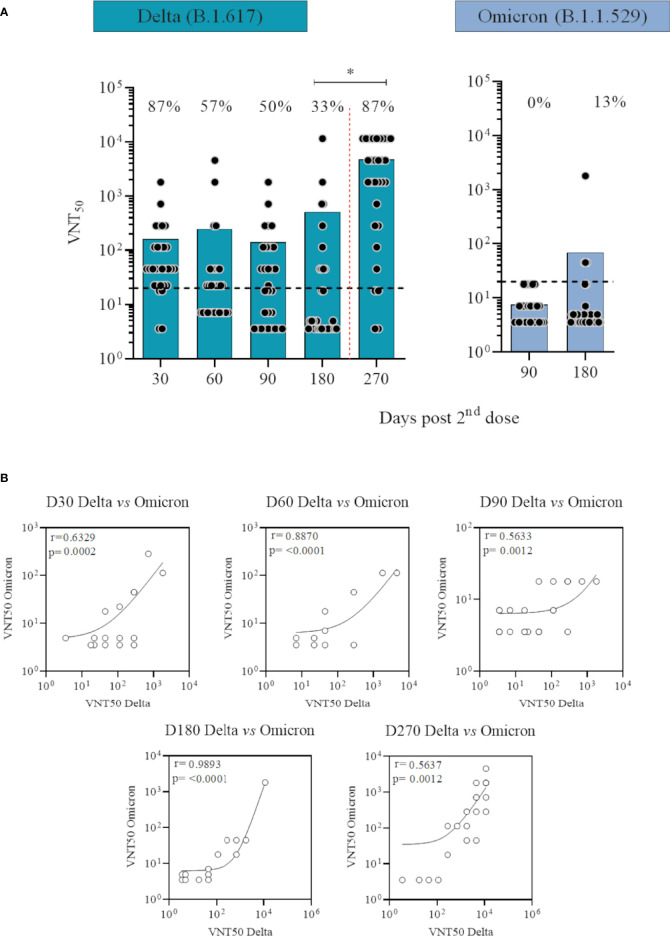
Neutralising antibody levels against Delta (B.1.617) and Omicron (B.1.1.529) SARS-CoV-2 variants over time in participants vaccinated with CoronaVac two-dose regimen plus BNT162b2 booster without previous COVID-19. **(A)** Logged values from individual specimens are presented here grouped into number of days after second dose. Average of VNT50 (neutralising titers capable to inhibit 50% of SARS-CoV-2 cytopathic effect) values are represented by bars. A cut off at 20 (black dotted line) is shown. Red dotted lines represent booster dose timepoint. ANOVA statistical differences are presented by letters for comparisons over timepoints for the same variant. **(B)** Correlation between neutralising antibodies titers against Delta and Omicron variants over time. Spearman rho and p values are shown.

Significant differences were seen for infectious SARS-CoV-2 microneutralisation titers achieved 30 and 60 days after CoronaVac in response to Delta (**Figure 3A**; **Supplementary Table 3**) and Omicron variants (**Supplementary Table 3**) (p=0·0002 and p=0·0048, respectively for the two timepoints) on non-infected individuals when comparing to our previous published data (11). Two doses of CoronaVac induced neutralising antibodies in 87% and 57% of participants against Delta variant, respectively in 30 and 60 days. Six months after CoronaVac vaccination and before the booster with BNT162b2, 33% of the population had detectable neutralising antibodies against Delta, versus 13% against Omicron. Average neutralisation titers peaked after the booster dose of BNT162b2 (p<0·0001), with an 87% seropositivity rate against Delta, re-establishing seropositivity rate generated by 2 doses of CoronaVac in the first 30 days for this variant. Neutralisation titers were below the lower limit of detection 90 days in VNT assays using Omicron variant compared with 50% responders at the same timepoint against Delta. There was a statistically significant degree of correlation between the neutralising antibodies titers against Delta and Omicron variants throughout the study with a Spearman rho of 0·6329, 0·8870, 0·5633, 0·9893 and 0·5637, respectively for the five timepoints (p<0·0012) (**Figure 3B**). Individuals presenting neutralising antibodies against Omicron presented the highest titers against Delta. There was also a correlation between the concentrations of the anti-spike IgG and the neutralising antibodies against both variants (p<0·05) for all timepoints, with one exception 30 days after second dose (p=0·8643 and 0·2185, respectively for Delta and Omicron correlation) (**Supplementary Figure 1**).

**Figure 4:**
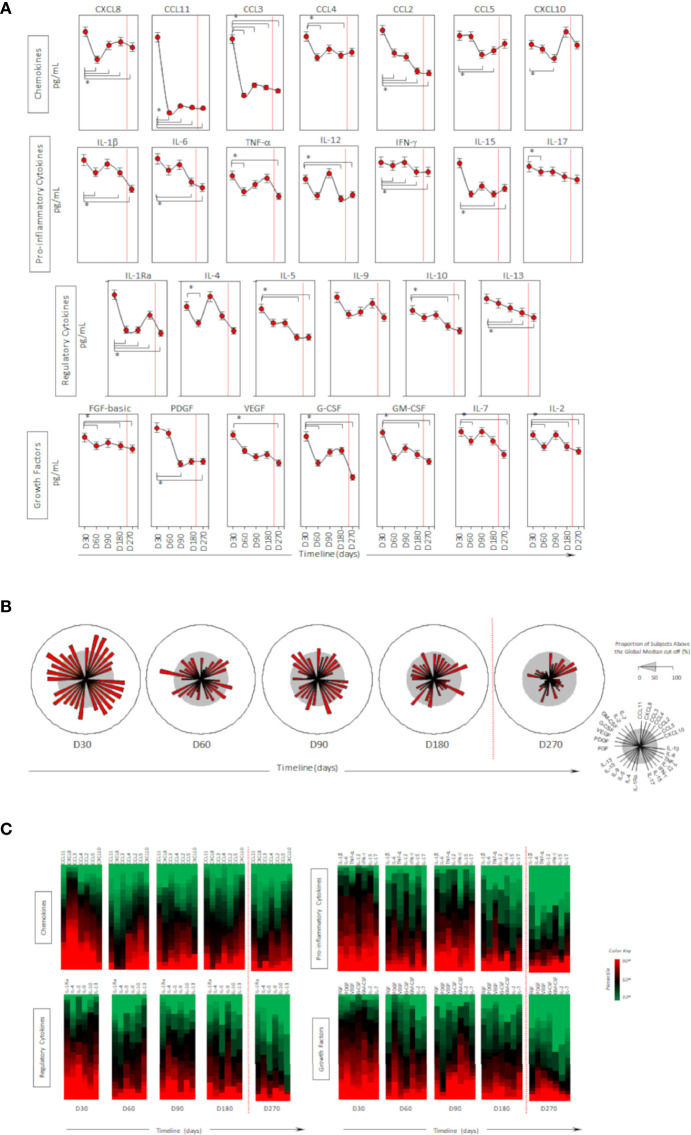
Timeline kinetics of serum biomarkers in participants vaccinated with CoronaVac two-dose regimen plus BNT162b2 booster dose. Cross-sectional follow-up analysis of levels of chemokines (CXCL8, CCL11, CCL3, CCL4, CCL2, CCL5, CXCL10); pro-inflammatory cytokines (IL-1β, IL-6, TNF-α, IL-12, IFN-γ, IL-15, IL-17); regulatory cytokines (IL-1Ra, IL-4, IL-5, IL-9, IL-10, IL-13) and growth factors (FGF-basic, PDGF, VEGF, G-CSF, GM-CSF, IL-7, IL-2) was carried out in serum samples. Measurements were performed at distinct times post second dose protocol (30-, 60-, 90-, 180-, and 270-days). Biomarkers were presented individually in multiple subpanels and displayed in classes: chemokines, pro-inflammatory cytokines, regulatory cytokines, and growth factors. Red dotted lines represent the booster dose timepoint. In **(A)** timeline kinetics; **(B)** overall signature of mediators; and **(C)** heatmap constructs. ANOVA statistical differences are presented by the letter for comparisons over timepoints (*).

**Figure 5:**
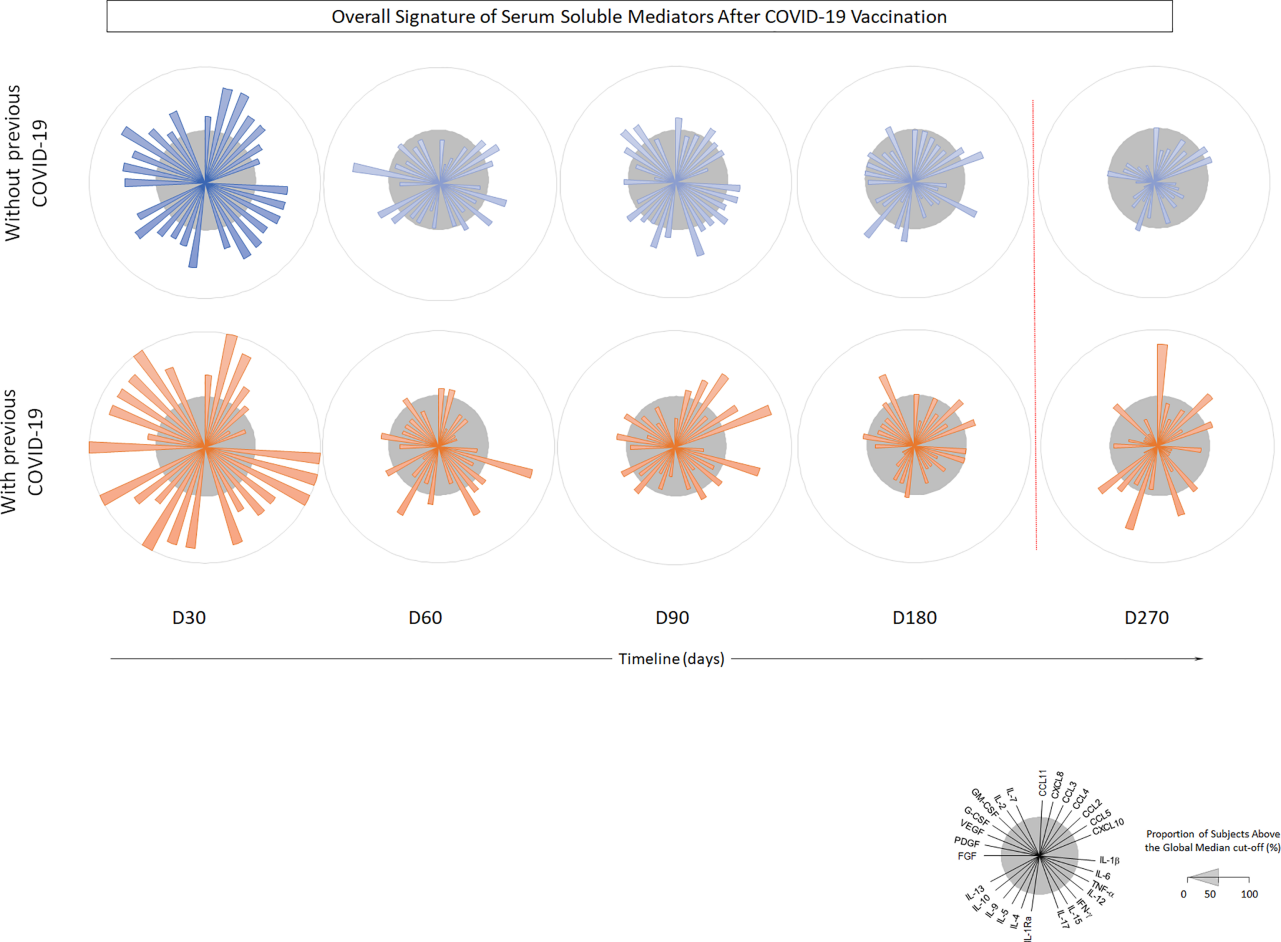
Timeline kinetics of serum biomarkers in participants with or without COVID-19 prior to vaccination with CoronaVac 2 doses + BNT162b2 booster dose. Cross-sectional follow-up analysis of chemokines (CXCL8, CCL11, CCL3, CCL4, CCL2, CCL5, CXCL10); pro-inflammatory cytokines (IL-1β, IL-6, TNF-α, IL-12, IFN-γ, IL-15, IL-17); regulatory cytokines (IL-1Ra, IL-4, IL-5, IL-9, IL-10, IL-13) and growth factors (FGF-basic, PDGF, VEGF, G-CSF, GM-CSF, IL-7, IL-2) was carried out in serum samples. Measurements were performed at distinct times after primary protocol (30-, 60-, 90-, 180- and 270-days post second dose). Biomarkers were presented individually in multiple subpanels and displayed in classes: chemokines, pro-inflammatory cytokines, regulatory cytokines, and growth factors. Red dotted lines represent booster dose timepoint.

**Figure 6:**
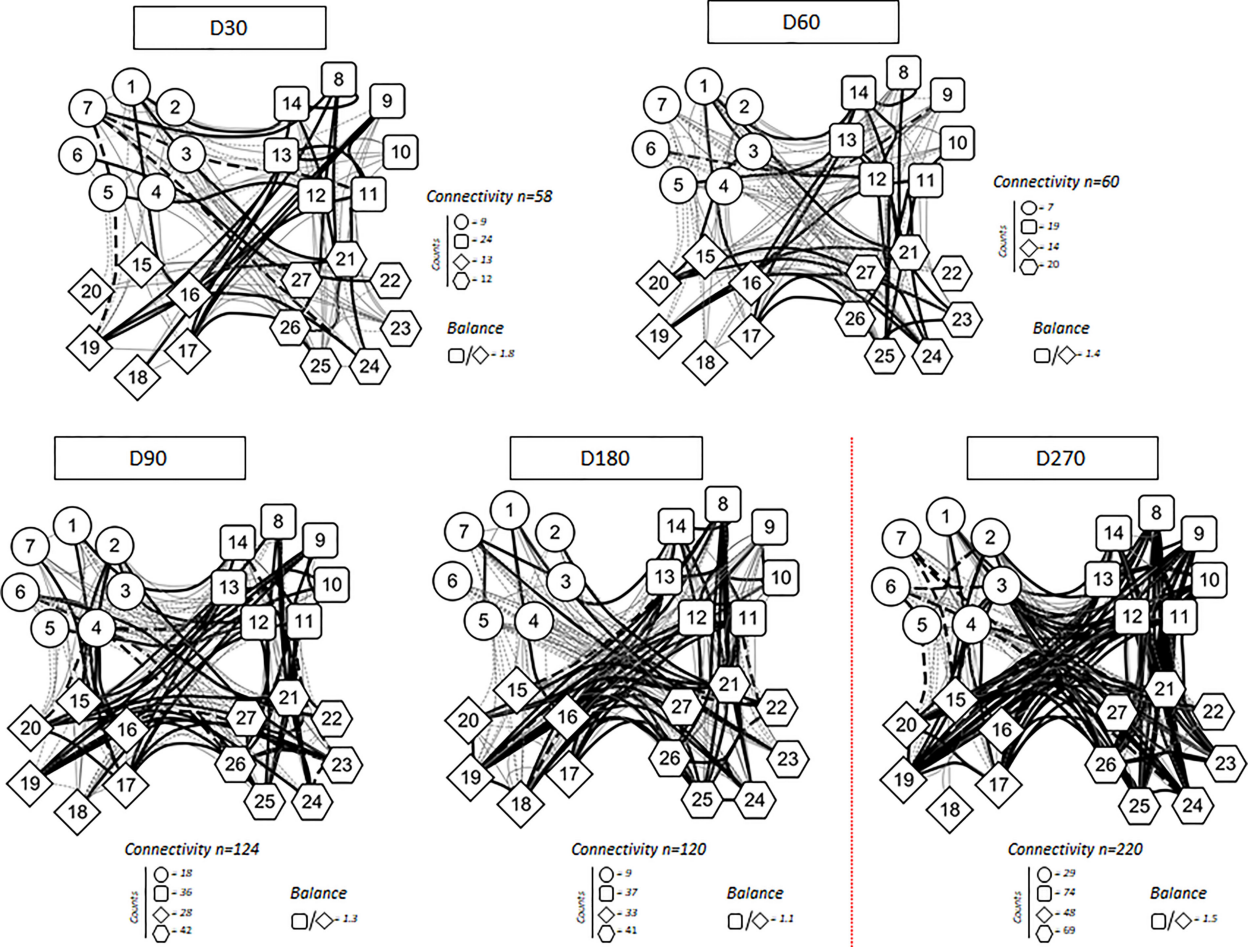
Correlation analysis presented as a network of immunological chemokines (O), pro-inflammatory cytokines (□), regulatory cytokines (◊), and growth factors (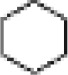) over the time after vaccination. Each parameter is shown in a node. Red dotted lines represent booster dose timepoint. Statistical analysis was performed using the Spearman correlation test and the significant correlations (p < 0.05) are represented by a line connecting both nodes. The correlation was classified as weak (r < 0), moderate (0.36 < r < 0.68) and strong (r > 0.68), based on absolute value of correlation index r, represented by line thickness. Thick and thin connecting edges represent strong and moderate correlation between soluble mediators, respectively. Positive correlation is expressed by a continuous line, while negative correlation by dashed lines. Number of connectivity and balance between pro-inflammatory and regulatory cytokine are expressed.

Higher levels of chemokines (CXCL8, CCL2, CCL3, CCL4, CCL11, CXCLl0), pro-inflammatory cytokines (IL-1β, IL-6, TNF-a, Il-15, IL-17), regulatory cytokines (IL-1Ra, IL-5, IL-9, IL-10, IL-13) and growth factors (FGF-basic, PGGF, VEGF, G-CSF and GM-CSF) were found 30 days after CoronaVac vaccination, when compared to subsequent timepoints. Indeed, all the vast majority of the soluble mediators’ levels was reduced over time and no increase after the BNT162b2 booster was observed (**Figures 4A–C**). Separate analyses of the immune response generated in individuals who had COVID-19 prior to vaccination demonstrated greater expansion of soluble mediators in the initial 30-day period compared to those who did not have the previous COVID-19. Over the one-year period, these individuals maintained high values of some mediators, with a higher expansion after the booster dose (**Figure 5**). Despite the reduction of mediators over time, an increase of correlation of those molecules over time after CoronaVac vaccination was observed, with a stronger interaction after the introduction of the heterologous booster dose (**Figure 6**). The connectivity between mediators was highlighted by the balance of pro-inflammatory and regulatory cytokines related to a mixed pro-inflammatory and regulatory profile. A reduction of this balance was demonstrated over time; however it was restored after the booster dose, showing a main interaction of TNF-a, IFN-g, IL-1β, 1Ra, IL-4, IL-5, IL-6, IL-9, IL-10, IL-12, IL-13, IL-15, IL-17.

### Reactogenicity

**Figure 7:**
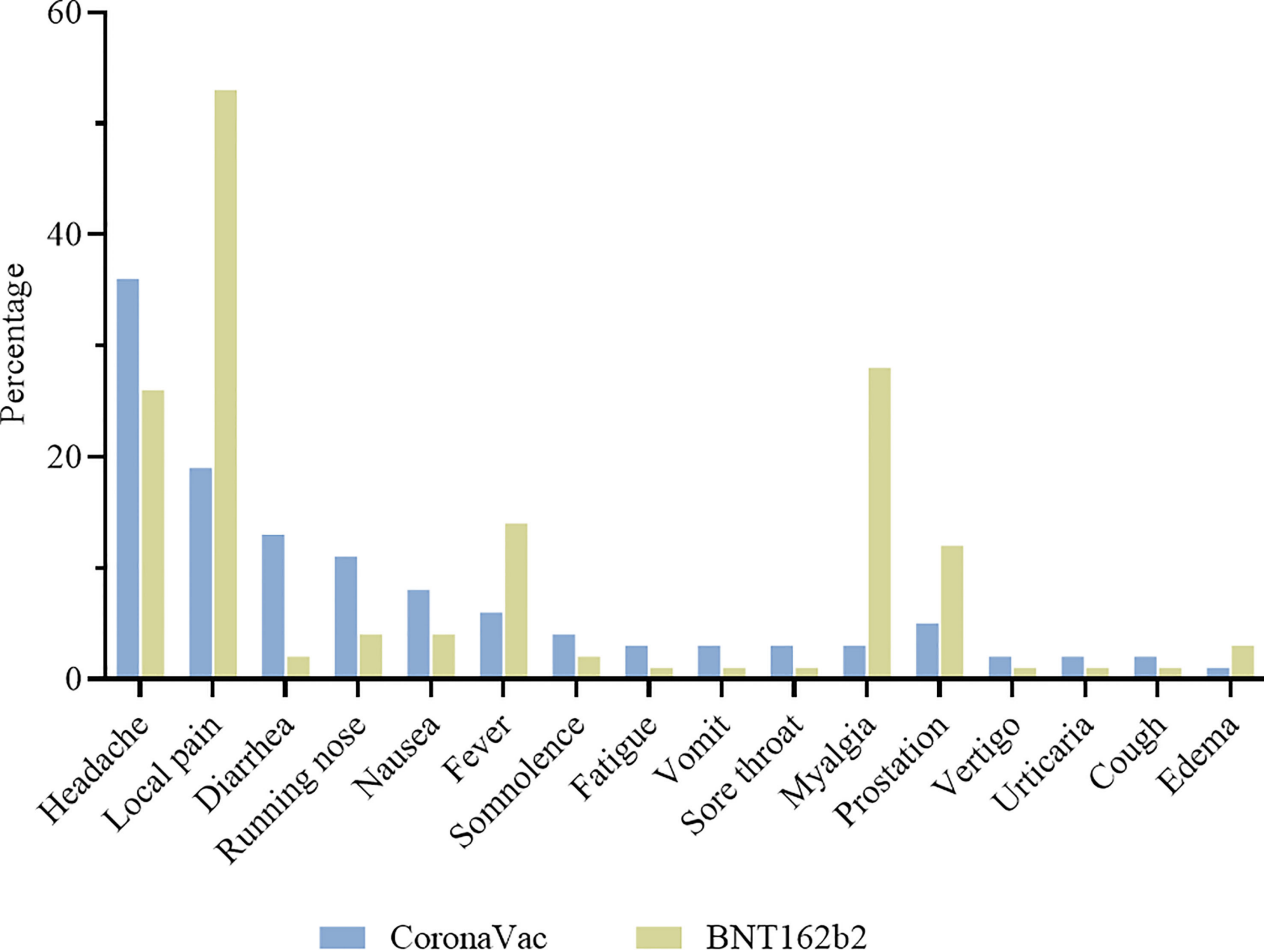
Adverse events in the first 7 days after vaccination. Participants were vaccinated with CoronaVac two-dose regimen and BNT162b2 booster dose. Most frequent adverse events are described for CoronaVac (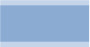) and BNT162b2 (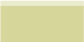).

A total of 325/1587 (20·5%) and 391/736 (53·1%) participants presented some adverse event after CoronaVac and BNT162b2 vaccination, respectively. The most common vaccine reactions were headache experienced by 36% of those receiving CoronaVac and 26% for BNT162b2; and injection site pain by 19% for CoronaVac and 53% of those receiving BNT162b2. Diarrhea was common for CoronaVac (13%), compared with 2% for BNT162b2. Running nose was also commonly reported, in 11% and 4% of CoronaVac and BNT162b2 respectively. Fever, myalgia, and prostration were common for BNT162b2 (14%, 28% and 12%), but not for recipients of CoronaVac (6%, 3% and 5%) (**Figure 7**).

Five moderate adverse events were recorded, although it was not possible to correlate them with the vaccines received. Seven CoronaVac vaccinated participants described dyspnea (4), desaturation (2) and hypertension (1) in the first 48 hours after the second dose. Five participants described hypertension (3), mental confusion (1) and fainting (1) in 24 hours after BNT162b2 booster dose. All participants recovered and no serious adverse event was recorded.

### Effectiveness

**Figure 8:**
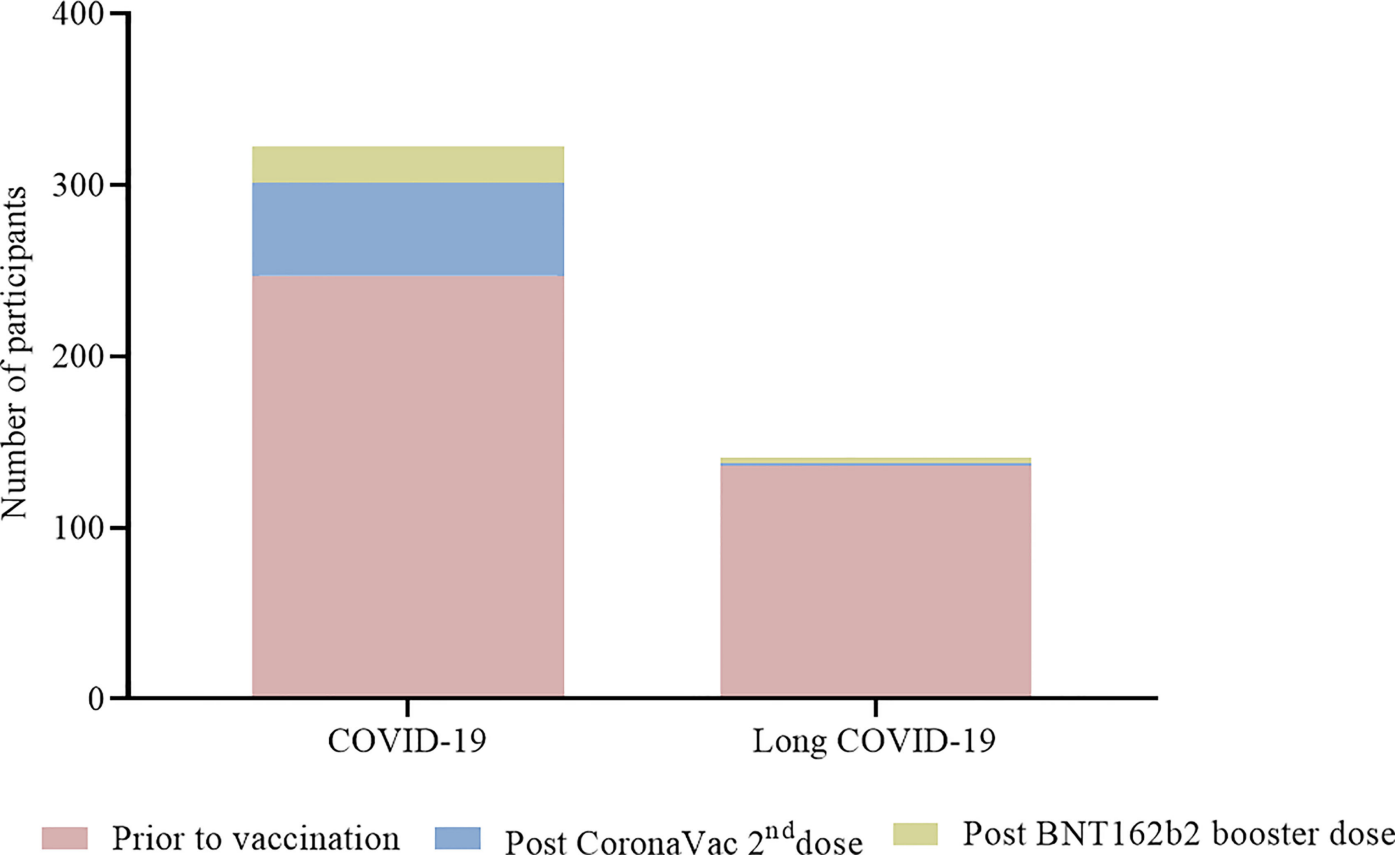
Participants with COVID-19 and long COVID before and after vaccination. Numbers of participants are presented in each column for participants before vaccination (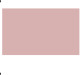), vaccinated with two doses of CoronaVac only (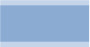) and vaccinated with two doses of CoronaVac + BNT162b2 booster dose (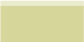).

Among the 1,587 vaccinated individuals, a total of 247 (15·6%) had a positive RT-qPCR before vaccination. Among them, 136 (8·6%) were diagnosed with long COVID, reporting remaining symptoms after COVID-19 in subsequent months. Two hundred sixty-one individuals had suspected symptoms and were tested by RT-qPCR. A decrease of COVID-19 was observed after vaccination with 75 (4·7%) RT-qPCR positive individuals among which 5 (0·3%) developed long COVID (**Figure 8**) with mild persistent symptoms for at least 3 months. Sequencing of 29 samples obtained from vaccinated individuals revealed 13 Delta, 12 Omicron, 3 gamma and 1 alpha infections (**Supplementary Table 4**).

## Discussion

SARS-CoV-2-specific humoral and cellular responses were analyzed for a year period in individuals who underwent vaccination with two doses of CoronaVac and booster with BNT162b2 in Brazil after 6-months of primary protocol. We observed that anti-Spike IgG titers declined 80 days after second dose and increased 1.7 times after the introduction of the BNT162b2 booster dose in comparison to the prior timepoint, leading to seropositivity of 100% in 15 days post booster. The significant increase in humoral response seen with heterologous boosting in our study reflects similar findings from other trials such as the RHH-001 and a Turkish study which compared homologous and heterologous boosting after CoronaVac primary protocol (16, 17). Although levels of protection are expected to begin to reduce over time, like some other vaccines, it is important to mention that individuals who did not receive a booster dose remained 74% seropositive even after a year. Similar pattern on anti-Spike IgG kinetics was seen for all age groups, although seropositivity in older adults (61-90 years) was 15% lower than in younger adults at days 150-200 post CoronaVac vaccination and then restored after BNT162b2 booster. Other studies demonstrated that CoronaVac effectiveness against death by COVID-19 is higher from younger to elder decades of life, with 84·8% in those < 60 years compared to 63·5% of vaccinees aged 80–89 years and 48·6% for individuals aged ≥ 90 years (18, 19). Prior COVID-19 generated an increased seropositivity rate throughout the analyzed period with a rise in anti-Spike IgG levels from 60 to 270 days post CoronaVac vaccination that were superior to those induced by previously uninfected individuals. These data are supported by other findings (20).

With the arising of new SARS-CoV-2 variants, a discussion about booster doses and vaccine effectiveness was initiated. Some studies showed evidence of neutralising antibody titers fading against the new Omicron variant (21–23), although no evaluation was done for a longer period as we presented. Our data show that titers of neutralising antibodies to Omicron variant were lower in all timepoints than those found against Delta, especially after 30 and 60 days of CoronaVac vaccination (11). Two doses of CoronaVac induced neutralising antibodies in 87% and 17% of participants in 30 days and 33% and 13% 6 months after second dose, respectively against Delta and Omicron variants. Titers peaked after BNT162b2 booster dose, with an 87% seropositivity rate against Delta and 77% against Omicron (11). Correlation analyses showed that individuals presenting neutralising antibodies against Omicron also presented the highest titers against Delta and anti-Spike antibodies. If low neutralising antibody titer is related to a reduction on the immune protection, it must be further clarified as variants may evade antibody response but can be still well recognized by T cells.

This study showed, in an unprecedented level of detail, a robust release of soluble mediators of the cellular immune response in the first month after CoronaVac vaccination followed by a gradual reduction over time and no increase was seen after the BNT162b2 booster dose. Nonetheless, a stronger interaction between mediators was noted over time, and it became more robust after the introduction of the heterologous booster dose. The detection of a balance of pro-inflammatory and regulatory cytokines indicates a mixed immune profile induced by the two-dose regimen of CoronaVac and BNT162b2 booster. Individuals who had COVID-19 prior to vaccination demonstrated greater expansion of soluble markers over kinetics when compared to those who had no prior COVID-19, indicating that prior exposure to the virus leads to a more robust and multifunctional response. Higher values were seen in the initial 30 days after CoronaVac and again after the BNT162b2 booster dose. While studies show a progressive loss of humoral and cellular immune response in vaccines for viral infections (24–26) and a durable cellular immune response that persisted for at least 6 months after either mRNA-based or adenovirus vector-based vaccination against SARS-CoV-2 (27), we present pioneer data about cellular response generated by CoronaVac over a nine-months period.

CoronaVac and BNT162b2 were both safe and well tolerated among individuals. Although 20·5% and 53·1% of CoronaVac and BNT162b2 vaccinees, respectively, presented some adverse event, they were mild and transient. The most common vaccine reactions were headache and injection site pain. Diarrhea was more common for CoronaVac, whereas fever, myalgia, and prostration were commonly reported for BNT162b2. Our findings were similar to the results of others (16, 28).

Noteworthy, a significant percentage of individuals (15·6%) had a positive RT-qPCR before vaccination and 8·6% were diagnosed with long COVID, against 4·7% RT-qPCR positives after vaccination and 0·3% with long COVID. Indeed, long COVID is a complex and an increasingly recognized condition with secondary complications that are poorly understood (29). Beyond symptoms, people with long COVID are reporting impaired quality of life and impact on physical and cognitive functions (30).

Combined cellular and humoral immune response determine the clinical course of a viral infection and effectiveness of vaccination. This study brings pioneering contributions on the induction and maintenance of the immune response triggered by vaccination with two doses of CoronaVac and a BNT162b2 booster. While epidemiological data has been showing the overall vaccine effectiveness against SARS-CoV-2 infection and COVID-19 hospitalization waned over time (10), our study brings a complete evaluation of the immune response over time on real life basis, approaching the effectiveness of CoronaVac association with BNT162b2 from the clinical and biological perspectives, aspects that have important repercussions for informing decisions about the necessity of vaccine boosters.

## Data Availability Statement

Data that underlie the results reported in this article, after de-identification (text, tables, figures, and appendices), will be available for researchers who provide a methodologically sound proposal to the corresponding author. To gain access, data requestors will need to sign a data access agreement.

## Ethics Statement

Ethical approval was given to Immunita-001 study by the Ethical Review Committee (CAAE 2898621.9.0000.5091). The patients/participants provided their written informed consent to participate in this study.

## Immunita-001 Team

Priscila Fernanda S Martins, Maria Luysa C Pedrosa, MS, Jessica V de Assis, MS, Viviane CF dos Santos, PhD, Eduardo R de Oliveira, MS, Raphael A Silva, Maria Izabella VARC Medeiros, MS, Rafaela OV Coelho, Cecilia MF Bicalho, Raquel VR Vilela, PhD.

## Author Contributions

Conceived and designed the experiments: RG, NA, OM-F, MN, PA. Analyzed the data: RG, NA, OM-F, JO, AT-C, MN, PA, GF, AC-A. Wrote the paper: RG, NA. Performed experiments: PF, CC, SG, DM, AL, GC, LC, TL, DA, RA, TS, WJ, DO, AC-A. All authors contributed to the article and approved the submitted version.

## Funding

This study was funded by the Oswaldo Cruz Foundation (FIOCRUZ) to RG, the Innovative Products to face COVID-19 pandemics initiative (grant number VPPIS-005-FIO-20-2-45) to PA, and by the Brazilian Ministry of Science, Technology and Innovation (MCTI) through the “Rede Virus” initiative to PA (grant number – FINEP 01.20.0005.00). NA is supported by Brazilian Federal Agency for Support and Evaluation of Graduate Education (CAPES) (CAPES-PrInt), PF is supported by the Minas Gerais Research Foundation (FAPEMIG), CA-C is supported by FIOCRUZ, SG and DM are supported by the Brazilian National Council for Scientific and Technological Development (CNPq). AC and OM-F received PQ fellowships from CNPq and are research fellows from FAPEAM (PVN-II, PRÓ-ESTADO Program #005/2019). GC is supported by FAPESP (2020/07419-0); MN is partly funded by the Centers for Research in Emerging Infectious Diseases (CREID), “The Coordinating Research on Emergent Arboviral Threats Encompassing the Neotropics (CREATE-NEO)” grant U01 AI151807 by the National Institutes of Health (NIH/USA). MLN is supported by a FAPESP COVID Grant (2020/04836-0); MN is a Brazilian National Council for Scientific and Technological Development (CNPQ) Research Fellow.

## Conflict of Interest

MN has received research grants from Instituto Butantan, Janssen Vaccines and Prevention B.V., Medicago R&D Inc, and Pfizer/BioNTech SE.

The remaining authors declare that the research was conducted in the absence of any commercial or financial relationships that could be construed as a potential conflict of interest.

## Publisher’s Note

All claims expressed in this article are solely those of the authors and do not necessarily represent those of their affiliated organizations, or those of the publisher, the editors and the reviewers. Any product that may be evaluated in this article, or claim that may be made by its manufacturer, is not guaranteed or endorsed by the publisher.
