# Supplementary material for: Immunogenicity, Effectiveness, and Safety of Inactivated Virus (CoronaVac) Vaccine in a Two-Dose Primary Protocol and BNT162b2 Heterologous Booster in Brazil (Immunita-001): A One Year Period Follow Up Phase 4 Study
Source: Front Immunol. 2022 Jun 9;13:918896. doi: 10.3389/fimmu.2022.918896 (PMC9218743; doi:10.3389/fimmu.2022.918896)
Supplement: Supplementary file 1 [file DataSheet_1.docx]

**
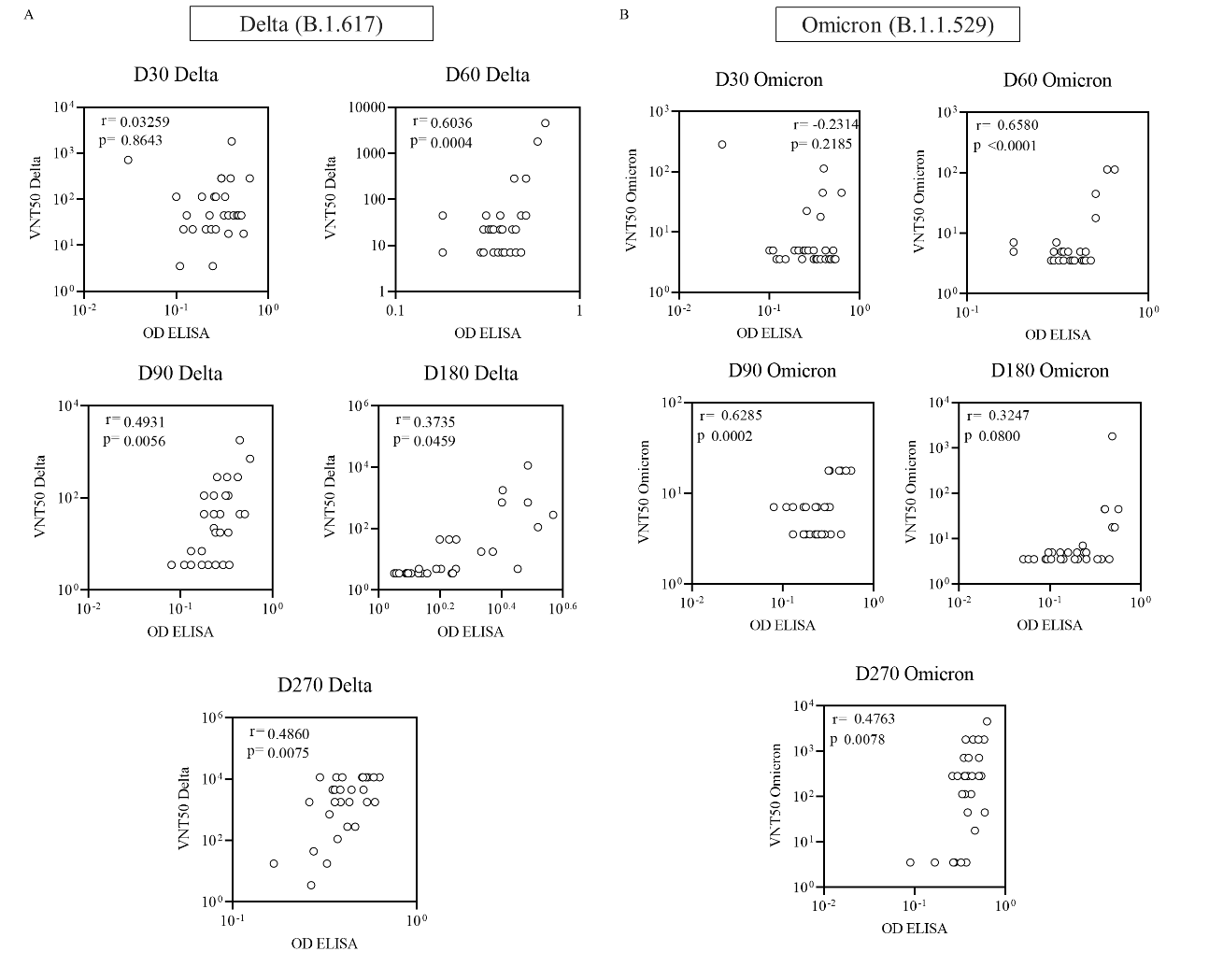
**

Supplementary Figure 1. Correlation between anti-spike IgG and neutralising antibodies titers against Delta (A) and Omicron (B) variants over time. Spearman rho and p values are shown.
