# Supplementary material for: Immunogenicity, Effectiveness, and Safety of Inactivated Virus (CoronaVac) Vaccine in a Two-Dose Primary Protocol and BNT162b2 Heterologous Booster in Brazil (Immunita-001): A One Year Period Follow Up Phase 4 Study
Source: Front Immunol. 2022 Jun 9;13:918896. doi: 10.3389/fimmu.2022.918896 (PMC9218743; doi:10.3389/fimmu.2022.918896)
Supplement: Supplementary file 2 [file Table_1.docx]

Supplementary table 1. Primer sequences added to the cDNA amplification step and sample names deposited in GISAID database.

| Primer Name | Sequence | Concentration |
| --- | --- | --- |
| COVIDSEQ_3732_FNF | 5’- GTTGTTAATGCAGCCAATGTTTACCTTAAA - 3’ | 20 nM |
| COVIDSEQ_4186_FNR | 5’- CAACTTGCTTTTCACTCTTCATTTCCAAA - 3’ |  |
| COVIDSEQ_20883_FNF 5' | 5’- TGCTAATTCCATTGTTTGTAGATTTGACACTA - 3’ |  |
| COVIDSEQ_21285_FNR | 5’- CTGAAGTCTTGTAAAAGTGTTCCAGAGG - 3’ |  |
| COVIDSEQ_24259_FNF | 5’- AACATCACTAGGTTTCAAACTTTACTTGCT - 3’ |  |
| COVIDSEQ_24604_FNR | 5’- ATGCAAATCTGGTGGCGTTAAAAAC - 3’ |  |

| **Sample ID** | **Lineage** |
| --- | --- |
| MG-FIOCRUZ-99 | P.1.7 |
| MG-FIOCRUZ-102 | P.1 |
| MG-FIOCRUZ-110 | B.1.1 |
| MG-FIOCRUZ-446 | P.1 |
| MG-FIOCRUZ-117 | AY.6 |
| MG-FIOCRUZ-448 | AY.99.2 |
| MG-FIOCRUZ-449 | AY.6 |
| MG-FIOCRUZ-121 | AY.6 |
| MG-FIOCRUZ-176 | AY.6 |
| MG-FIOCRUZ-177 | P.1 |
| MG-FIOCRUZ-179 | B.1.617.2 |
| MG-FIOCRUZ-180 | AY.6 |
| MG-FIOCRUZ-452 | AY.99.2 |
| MG-FIOCRUZ-454 | AY.99.2 |
| MG-FIOCRUZ-455 | AY.99.2 |
| MG-FIOCRUZ-547 | AY.43.1 |
| MG-FIOCRUZ-1056 | BA.1 |
| MG-FIOCRUZ-1057 | BA.1 |
| MG-FIOCRUZ-1058 | BA.1 |
| MG-FIOCRUZ-1059 | BA.1 |
| MG-FIOCRUZ-1060 | BA.1 |
| MG-FIOCRUZ-1061 | BA.1 |
| MG-FIOCRUZ-1062 | BA.1 |
| MG-FIOCRUZ-1063 | BA.1 |
| MG-FIOCRUZ-1064 | BA.1 |
| MG-FIOCRUZ-1065 | BA.1 |
| MG-FIOCRUZ-1066 | BA.1 |
| MG-FIOCRUZ-1067 | BA.1 |
