# Supplementary material for: Immunogenicity, Effectiveness, and Safety of Inactivated Virus (CoronaVac) Vaccine in a Two-Dose Primary Protocol and BNT162b2 Heterologous Booster in Brazil (Immunita-001): A One Year Period Follow Up Phase 4 Study
Source: Front Immunol. 2022 Jun 9;13:918896. doi: 10.3389/fimmu.2022.918896 (PMC9218743; doi:10.3389/fimmu.2022.918896)
Supplement: Supplementary file 3 [file Table_2.docx]

Supplementary Table 2. Subgroups of the study population included in the analysis of immunogenicity according to their age and previous COVID-19 status in each timepoint over one-year period.

| **Age** |  | **Day 30** | **Day 60** | **Day 90** | **Day 180** | **Day 270** | **Day 350** |
| --- | --- | --- | --- | --- | --- | --- | --- |
| All | N | 460 | 575 | 928 | 581 | 563 | 618 |
|  | Median | 0·330 | 0·340 | 0·310 | 0·222 | 0·260 | 0·456 |
|  | 95% Confidence Interval | 0·014 | 0·011 | 0·009 | 0·012 | 0·013 | 0·010 |
| 18-30 | N | 28 | 189 | 200 | 159 | 99 | 140 |
|  | Median | 0·095 | 0·348 | 0·354 | 0·230 | 0·291 | 0·456 |
|  | 95% Confidence Interval | 0·072 | 0·018 | 0·019 | 0·022 | 0·031 | 0·025 |
| 31-40 | N | 23 | 171 | 377 | 192 | 238 | 229 |
|  | Median | 0·070 | 0·350 | 0·300 | 0·226 | 0·230 | 0·455 |
|  | 95% Confidence Interval | 0·023 | 0·020 | 0·013 | 0·019 | 0·019 | 0·015 |
| 41-50 | N | 19 | 125 | 234 | 135 | 154 | 169 |
|  | Median | 0·090 | 0·350 | 0·294 | 0·218 | 0·264 | 0·457 |
|  | 95% Confidence Interval | 0·087 | 0·023 | 0·016 | 0·028 | 0·026 | 0·019 |
| 51-60 | N | 8 | 60 | 96 | 67 | 63 | 70 |
|  | Median | 0·095 | 0·329 | 0·319 | 0·204 | 0·272 | 0·459 |
|  | 95% Confidence Interval | 0·059 | 0·031 | 0·028 | 0·034 | 0·044 | 0·031 |
| 61-90 | N | 2 | 14 | 18 | 17 | 8 | 15 |
|  | Median | 0·070 | 0·340 | 0·314 | 0·186 | 0·157 | 0·471 |
|  | 95% Confidence Interval | 0·270 | 0·070 | 0·059 | 0·090 | 0·140 | 0·063 |
| **Previous COVID-19** |  |  |  |  |  |  |  |
| Yes | N | 12 | 89 | 159 | 73 | 96 | 83 |
|  | Median | 0·130 | 0·340 | 0·352 | 0·282 | 0·340 | 0·439 |
|  | 95% Confidence Interval | 0·136 | 0·031 | 0·021 | 0·032 | 0·034 | 0·032 |
| No | N | 68 | 470 | 766 | 497 | 466 | 540 |
|  | Median | 0·080 | 0·349 | 0·300 | 0·210 | 0·251 | 0·456 |
|  | 95% Confidence Interval | 0·032 | 0·011 | 0·009 | 0·013 | 0·014 | 0·010 |
