# Supplementary material for: Immunogenicity, Effectiveness, and Safety of Inactivated Virus (CoronaVac) Vaccine in a Two-Dose Primary Protocol and BNT162b2 Heterologous Booster in Brazil (Immunita-001): A One Year Period Follow Up Phase 4 Study
Source: Front Immunol. 2022 Jun 9;13:918896. doi: 10.3389/fimmu.2022.918896 (PMC9218743; doi:10.3389/fimmu.2022.918896)
Supplement: Supplementary file 4 [file Table_3.docx]

Supplementary Table 3. Neutralising antibody titers against Delta (B.1.617) and Omicron (B.1.1.529) SARS-CoV-2 variants in each timepoint over one-year period.

|  | **Day 30** | **Day 60** | **Day 90** | **Day 180** | **Day 270** |
| --- | --- | --- | --- | --- | --- |
| **Delta (B.1.617)** |  |  |  |  |  |
| N | 30 | 30 | 30 | 30 | 30 |
| Median | 44·90 | 22·27 | 20·05 | 4·95 | 3185·80 |
| 95% Confidence Interval | 126·17 | 322·78 | 127·81 | 773·09 | 1739·44 |
| **Omicron (B.1.1.529)** |  |  |  |  |  |
| N | 30 | 30 | 30 | 30 | 30 |
| Median | 4·95 | 4·25 | 7·07 | 4·25 | 285·09 |
| 95% Confidence Interval | 20·01 | 10·36 | 2·00 | 120·80 | 350·07 |

*Caption: Campos GRF, et al. 2022. doi: <https://doi.org/10.1101/2022.03.24.22272904>.
