# Supplementary material for: Immunogenicity, Effectiveness, and Safety of Inactivated Virus (CoronaVac) Vaccine in a Two-Dose Primary Protocol and BNT162b2 Heterologous Booster in Brazil (Immunita-001): A One Year Period Follow Up Phase 4 Study
Source: Front Immunol. 2022 Jun 9;13:918896. doi: 10.3389/fimmu.2022.918896 (PMC9218743; doi:10.3389/fimmu.2022.918896)
Supplement: Supplementary file 5 [file Table_4.docx]

Supplementary Table 4. RT-qPCR and sequencing results from the 29 COVID-19 positive individuals during the study.

| **Case** | **CT RNAse P** | **CT Gene E** | **SARS-CoV-2**  **RNA** | **Sequencing** |
| --- | --- | --- | --- | --- |
| 1 | 27.154 | 21.827 | Detected | P.1 (K417T/E484K/N501Y) |
| 2 | 26.343 | 22.254 | Detected | P.1 (K417T/E484K/N501Y) |
| 3 | 27.525 | 23.16 | Detected | B.1.1.7 (N501Y) |
| 4 | 29.87 | 26.918 | Detected | Gamma (P.1) |
| 5 | 25.443 | 18.687 | Detected | Delta (L452R/T478K) |
| 6 | 29.942 | 27.973 | Detected | Delta (AY.99.2) |
| 7 | 27.359 | 28.964 | Detected | Delta (AY.6) |
| 8 | 29.976 | 21.885 | Detected | Delta (AY.6) |
| 9 | 26.372 | 21.598 | Detected | Delta (L452R/T478K/D614G) |
| 10 | 26.23 | 20.336 | Detected | Delta (L452R/T478K/D614G) |
| 11 | 24.805 | 16.437 | Detected | Delta (T478K/D614G) |
| 12 | 27.781 | 23.178 | Detected | Delta (L452R/T478K/D614G) |
| 13 | 26.705 | 25.841 | Detected | Delta (AY.99.2) |
| 14 | 27.178 | 19.203 | Detected | Delta (L452R/T478K/D614G) |
| 15 | 27.096 | 20.309 | Detected | Delta (AY.99.2) |
| 16 | 29.502 | 27.967 | Detected | Delta (AY.99.2) |
| 17 | 26.521 | 21.727 | Detected | Delta (L452R/T478K/D614G) |
| 18 | 28.286 | 22.744 | Detected | Omicron (BA.1-like) |
| 19 | 27.094 | 24.766 | Detected | Omicron (BA.1-like) |
| 20 | 28.984 | 27.622 | Detected | Omicron (BA.1-like) |
| 21 | 26.149 | 21.585 | Detected | Omicron (BA.1-like) |
| 22 | 29.915 | 23.737 | Detected | Omicron (BA.1-like) |
| 23 | 31.301 | 25.923 | Detected | Omicron (BA.1-like) |
| 24 | 31.321 | 28.760 | Detected | Omicron (BA.1-like) |
| 25 | 23.463 | 21.927 | Detected | Omicron (BA.1-like) |
| 26 | 29.394 | 24.102 | Detected | Omicron (BA.1-like) |
| 27 | 27.107 | 19.460 | Detected | Omicron (BA.1-like) |
| 28 | 30.113 | 29.229 | Detected | Omicron (BA.1-like) |
| 29 | 26.891 | 27.189 | Detected | Omicron (BA.1-like) |
